# Bacterial Communities Are Less Diverse in a Strepsipteran Endoparasitoid than in Its Fruit Fly Hosts and Dominated by *Wolbachia*

**DOI:** 10.1007/s00248-023-02218-6

**Published:** 2023-04-27

**Authors:** Sharon Towett-Kirui, Jennifer L. Morrow, Shannon Close, Jane E. Royer, Markus Riegler

**Affiliations:** 1 Hawkesbury Institute for the Environment, Western Sydney University, Locked Bag 1797, Penrith, NSW 2751 Australia; 2 Queensland Department of Agriculture and Fisheries, EcoSciences Precinct, Boggo Road, Dutton Park, QLD 4102 Australia

**Keywords:** Strepsiptera, Parasitisation, *Wolbachia*, *Dipterophagus daci*, Tephritid fruit fly, Microbiome

## Abstract

Microbiomes play vital roles in insect fitness and health and can be influenced by interactions between insects and their parasites. Many studies investigate the microbiome of free-living insects, whereas microbiomes of endoparasitoids and their interactions with parasitised insects are less explored. Due to their development in the constrained environment within a host, endoparasitoids are expected to have less diverse yet distinct microbiomes. We used high-throughput 16S rRNA gene amplicon sequencing to characterise the bacterial communities of *Dipterophagus daci* (Strepsiptera) and seven of its tephritid fruit fly host species*.* Bacterial communities of *D. daci* were less diverse and contained fewer taxa relative to the bacterial communities of the tephritid hosts. The strepsipteran’s microbiome was dominated by Pseudomonadota (formerly Proteobacteria) (> 96%), mainly attributed to the presence of *Wolbachia*, with few other bacterial community members, indicative of an overall less diverse microbiome in *D. daci*. In contrast, a dominance of *Wolbachia* was not found in flies parasitised by early stages of *D. daci* nor unparasitised flies*.* Yet, early stages of *D. daci* parasitisation resulted in structural changes in the bacterial communities of parasitised flies. Furthermore, parasitisation with early stages of *D. daci* with *Wolbachia* was associated with a change in the relative abundance of some bacterial taxa relative to parasitisation with early stages of *D. daci* lacking *Wolbachia*. Our study is a first comprehensive characterisation of bacterial communities in a Strepsiptera species together with the more diverse bacterial communities of its hosts and reveals effects of concealed stages of parasitisation on host bacterial communities.

**Supplementary Information:**

The online version contains supplementary material available at 10.1007/s00248-023-02218-6.

## Introduction 

Insects have associations with diverse microbial communities that are important in host biology, host fitness and immunity and can provide protection against pathogens, parasitoids and toxins [1–3]. Symbiotic microbes can reside within the digestive tract, in particular the gut lumen [4, 5], on the surface of the insect host (ectosymbionts) and within host cells and tissues (endosymbionts) [6, 7]. The most common endosymbionts are maternally inherited *Wolbachia* (Alphaproteobacteria) that occur in over 50% of insects and other arthropod species [8, 9]. In many hosts, they manipulate host reproduction to enhance the production of infected females and thereby their prevalence in populations [9, 10]. For example, the induction of cytoplasmic incompatibility (CI) results in embryonic mortality when infected males mate with uninfected females or females infected with an incompatible *Wolbachia* strain; however, this CI is overcome in embryos of females infected with the same or a compatible *Wolbachia* strain, which can rescue the CI effect [9, 10]. Besides reproductive manipulation, *Wolbachia* strains may be beneficial to their hosts. Some strains confer protection to their hosts against parasites, viruses and other pathogens [11–13]. Other *Wolbachia* strains can synthesise vitamins deficient in host diets; for instance *Wolbachia* provides B vitamins to the bedbug, *Cimex lectularius* [14, 15]. *Wolbachia* can also influence the microbiome of hosts [16–18]. For instance, *Wolbachia* alters the relative abundance of bacterial taxa within microbial communities in the parasitoid wasp *Nasonia vitripennis* [18], the cabbage root fly *Delia radicum* [19] and adult mosquitoes [16]*.* Conversely, other symbiotic bacteria can influence *Wolbachia* prevalence and abundance, for example, *Asaia* can impede the establishment and stable transmission of *Wolbachia* in mosquitoes [20].

Many studies of host-microbe interactions have been performed on free-living insects, but less so on parasites [21] such as endoparasitoids that develop entirely within free-living insects. So far, the microbiomes of Strepsiptera, an endoparasitic insect order, have not yet been investigated [22], likely due to its extraordinary characteristic of extreme endoparasitism. Strepsiptera consists of 630 known species that parasitise hosts belonging to the seven insect orders Blattodea, Diptera, Hemiptera, Hymenoptera, Mantodea, Orthoptera and Zygentoma [23]. All strepsipteran species are obligate endoparasitoids and almost entirely complete their life cycle within their hosts [24, 25]. Adult strepsipterans display stark sexual dimorphism, with adult males that have external morphological features of a free-living adult insect, while the females are neotenic (lacking adult features) and fully endoparasitic within their hosts, except for adult females of one strepsipteran family, the Mengenillidae (suborder Mengenillidia) which are free-living [25, 26]. Parasitisation of the host occurs via the first instar larvae (planidia), which leave a parasitised host to then enter a new host where they undergo hypermetamorphosis to the fourth larval instar [26, 27]. In Mengenillidae, the fourth larval instars of both sexes leave and pupate on the outside of their hosts, while in all other families (all contained within the suborder Stylopidia), both male pupae and neotenic females extrude through the hosts’ cuticle [26, 27]. Males then emerge from the pupae within the host while females remain fully endoparasitic. Therefore, parasitisation by strepsipterans becomes visible as stylopisation in the later stages of strepsipteran development, while early stages of Strepsiptera may remain concealed in hosts and may only be detected by dissection or molecular tools [28]. Furthermore, strepsipterans are koinobionts which means that the hosts continue to live and feed while these endoparasitoids develop within their insect host [26].

Host-parasite interactions can be shaped by microbes associated with either the host or the parasite [21, 29]. Microbes can protect their hosts against parasites, for instance, a bacterial symbiont of aphids, *Hamiltonella defensa*, can protect its hosts against the parasitoid wasp *Lysiphlebus fabarum* [30, 31]. In contrast, the host microbiome can also aid in the establishment of parasites in their insect hosts, as seen in the interaction between the tapeworm *Hymenolepis diminuta* and its intermediate host, the grain beetle *Tenebrio molitor* [32]*.* Strepsiptera have an intimate relationship with their hosts and depend entirely on their hosts for nourishment [33]. Therefore, endoparasitoids may compete with the hosts’ microbiota for resources. Furthermore, host-associated microbes can also influence host immunity. For instance, altering the bacterial communities of the fruit fly *Drosophila melanogaster* by antibiotic treatment influenced its resistance to the parasitoid wasp *Asobara tabida* by moderating the encapsulation rate of the parasitoid eggs by the host [34]. Conversely, parasitisation can affect the host microbiome, such as seen in larvae of the two moth species *Diatraea saccharalis* and *Spodoptera frugiperda* parasitised by the parasitoid wasp *Cotesia flavipes*, which changed the bacterial community composition and structure of the moth larvae [35].

While some host species may have few or no microbial associations [36], parasites and parasitoids may have less diverse microbiomes than their hosts due to the relatively small size, their life cycle and exclusive dependence on their hosts for resources. For example, a parasitic plant, the obligate parasite *Orobanche hederae*, exhibits a reduced microbiome compared to its host plant *Hedera* [37]. Similarly, the bacterial alpha diversity is lower within the intestinal tapeworm *Eubothrium* than in its host, the Atlantic salmon [38], and the microbiomes associated with entomopathogenic nematodes used in the biological control of insect pests are of relatively low complexity [39]. Yet, an amplicon sequencing study of one of the smallest insects, the parasitoid wasp *Megaphragma amalphitanum*, has revealed that it still carries a diverse variety of bacteria, albeit different in composition from other larger parasitoid wasp species [40].

Strepsipteran neotenic females reproduce viviparously and obtain nutrients exclusively from the host hemolymph [33]. Strepsipteran larvae have a gut, and nutrient uptake from the host hemolymph occurs in the midgut; however, after extrusion of the females, the strepsipteran gut is degenerate and filled with hemolymph [41], and nutrient uptake from the host hemolymph occurs via a particular structure, the apron [42]*.*Therefore, due to the complete dependence of Strepsiptera on their hosts, it could be predicted that they have a less diverse microbiome. Such microbiome simplicity in parasites may be a parallel feature to the reduced morphological and genomic characteristics observed in several parasitic, ectosymbiotic and endosymbiotic organisms [26, 43–45] including the reduced genomic characteristics exhibited by bacterial endosymbionts [46, 47].

Our study focused on *Dipterophagus daci*, a strepsipteran endoparasitoid of tephritid fruit flies [48] belonging to the family of Halictophagidae [28]. To date, *D. daci* is the only described strepsipteran endoparasitoid of Diptera (besides other undescribed strepsipteran endoparasitoids of platystomatid flies from Papua New Guinea) and has been reported from 22 species of the tephritid subfamily of Dacini in Australia and the Solomon Islands [28, 48, 49]. A recent study revealed that the presence of two *Wolbachia* strains previously detected in flies of seven Australian tephritid species [50, 51] was due to concealed parasitisation of these flies with early developmental stages of *D. daci* [28]. This recent study also concluded that *D. daci* is the actual host of the two *Wolbachia* strains *w*Ddac1 and *w*Ddac2, which occur at high prevalence in this host, and *D. daci* without *Wolbachia* is found in only about 10% of parasitised flies [28]. The two strains *w*Ddac1 and *w*Ddac2 belong to the *Wolbachia* supergroup A and have previously been characterised using the *Wolbachia surface protein* (*wsp*) gene and five multi locus sequence typing (MLST) loci [50].

Tephritid fruit fly species are diverse and can infest diverse host plants but also different plant parts [52, 53]. Furthermore, tephritids have bacterial communities that can vary in diversity and structure depending on life stage, fly species and phylogeny, host plant species, diet and rearing environment [54–56]. For example, the bacterial communities of the island fly, *Dirioxa pornia*, differs from those of *Bactrocera* species, and this could be due to their different life histories [54]. Furthermore, bacterial communities with diverse compositions were observed among different *Bactrocera* species suggesting that several factors such as host plant specialisation and domestication play a role in shaping the microbiome of tephritid fruit flies [54].

Our study aimed to explore the diversity and composition of bacterial communities of the strepsipteran *D. daci.* We hypothesised that due to its endoparasitic life cycle, the bacterial communities of *D. daci* consist of only few taxa and are distinct from the bacterial communities of its fruit fly hosts. Furthermore, we expected that *Wolbachia* would dominate the microbiome of *D. daci* but not of the parasitised fruit fly species. We also tested whether early stages of *D. daci* parasitisation influenced the fruit fly microbiome, and whether this was influenced by the presence of *Wolbachia* in *D. daci*. To address these questions, we performed high-throughput next generation amplicon sequencing analyses of the commonly used and conserved bacterial marker gene, the 16S rRNA gene, of (i) *D. daci* male pupae, (ii) fruit flies parasitised by early stages of *Wolbachia*-positive *D. daci*, (iii) fruit flies parasitised by early stages of *Wolbachia*-negative *D. daci* and (iv) unparasitised fruit flies.

## Materials and Methods

### Fruit Fly Collection and DNA Extraction

**Fig. 1:**
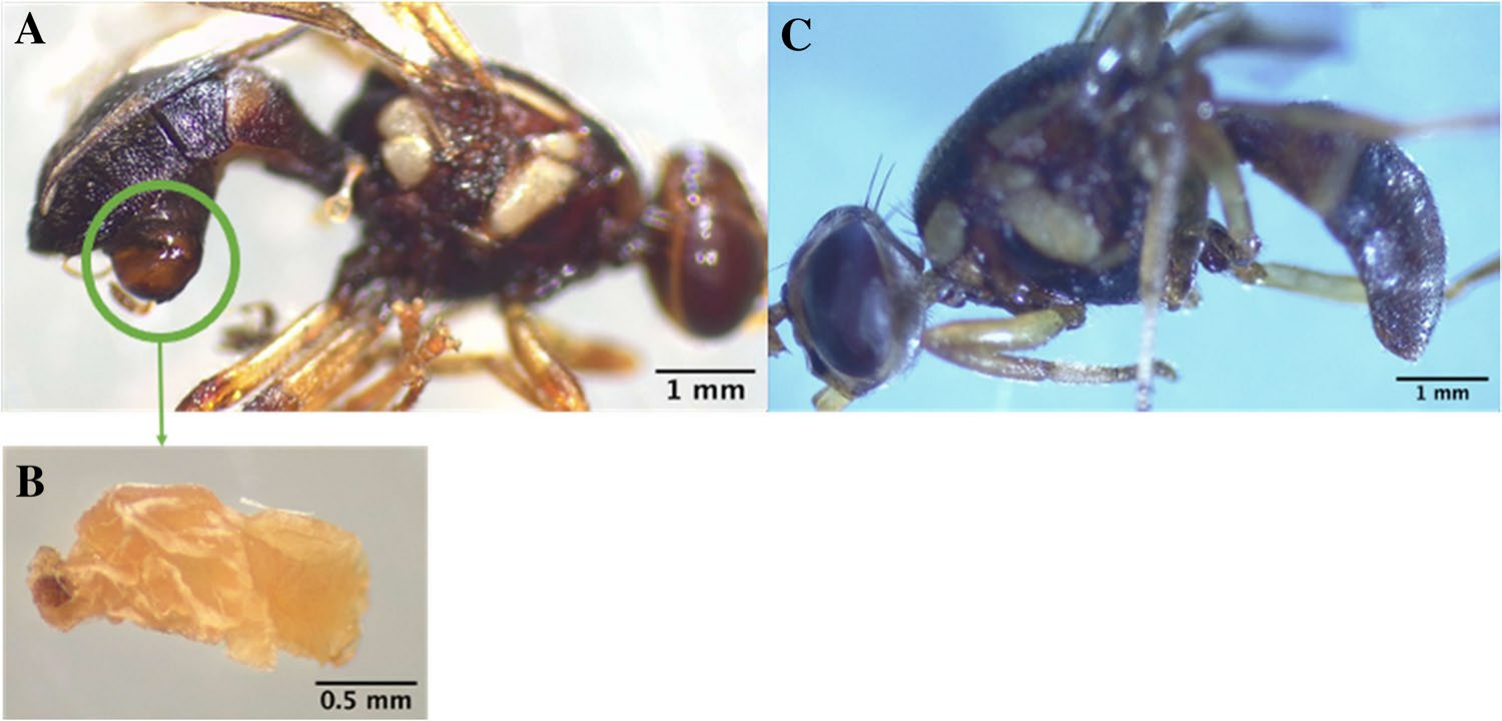
Field-caught male tephritid fruit flies collected using male lure traps. **A** Stylopised male fruit fly (*Bactrocera neohumeralis*); **B** *Dipterophagus daci* male pupa dissected from a stylopised male fruit fly (green circle shows the cephalotheca containing a male pupa); **C** non-stylopised male fruit fly (*Bactrocera bryoniae*)

We sequenced and analysed the bacterial 16S rRNA gene diversity of total genomic DNA extracts of 84 adult male fruit flies and 17 *D. daci* male pupae (Table S1) [28, 50, 51]. The 84 adult male fruit flies comprised individuals of seven species including *Bactrocera bryoniae* (*n* = 4), *Bactrocera decurtans* (*n* = 2), *Bactrocera frauenfeldi* (*n* = 11), *Bactrocera neohumeralis* (*n* = 22), *Bactrocera tryoni* (*n* = 32), *Dacus axanus* (*n* = 2) and *Zeugodacus strigifinis* (*n* = 11), collected from Queensland in 1998, 2001, 2012, 2013 and 2019 using male lure traps with malathion as part of fruit fly monitoring programs (Table S1) [57]. After emptying of traps, the trapped fruit fly specimens were kept dry and at room temperature for identification and then stored in ethanol at − 20 °C until DNA extraction. The *D. daci* male pupae were dissected from visibly parasitised (stylopised) male fruit flies of six species (*Bactrocera breviaculeus*, *B. frauenfeldi*, *Bactrocera mayi*, *Bactrocera pallida, B. neohumeralis* and *B. tryoni*; Table S1) collected from Queensland in 2019 [28]. The pupae were removed from the cephalotheca extruding from the abdomen of the parasitised fruit flies (Fig. 1a and b). In contrast to these stylopised individuals, the 84 adult male flies were either parasitised by concealed stages of *D. daci* (*D. daci*-positive by PCR) or unparasitised (*D. daci*-negative by PCR) (Fig. 1c) [28]. Prior to DNA extraction, the male fruit fly specimens and *D. daci* male pupae were surface-treated with 4% sodium hypochlorite to remove any external microorganisms and then washed with 0.2% Triton-X and rinsed thoroughly using Milli-Q water [51].

Total genomic DNA was extracted from individual fruit fly male abdomens and individual whole *D. daci* pupae using GenElute DNA Miniprep Kit (Sigma-Aldrich) as per the manufacturer’s instructions. The DNA quality was determined using NanoDrop and gel electrophoresis, and the extract was then stored at − 20 °C for subsequent experiments. The fruit fly and *D. daci* DNA extracts were screened by PCR using specific primers for the *wsp* and 16S rRNA genes [50, 51] and the *D. daci cytochrome c oxidase I* (*cox1*) gene [28]. Based on the PCR results, the samples were categorised into four groups: (i) *D. daci* male pupae (Dd) which were all positive for *Wolbachia* (*n* = 17), (ii) fruit flies parasitised by early stages of *Wolbachia*-positive *D. daci* (FliesDdW) (*n* = 30), (iii) fruit flies parasitised by early stages of *D. daci* without detectable *Wolbachia* (FliesDd) (*n* = 19) and (iv) unparasitised fruit flies (Flies) (*n* = 35) (Table S1). It is noted that *Wolbachia*-negative *D. daci* are relatively rare [28], and, therefore, flies parasitised by early stages of *D. daci* without detectable *Wolbachia* were preferentially included in our amplicon sequencing study to obtain a fair representation when compared to flies parasitised by early stages of *Wolbachia*-positive *D. daci*.

### Bacterial 16S rRNA Gene Amplification and Sequencing

The DNA extracts were submitted for 16S rRNA gene amplicon sequencing on an Illumina MiSeq platform at the Western Sydney University Next Generation Sequencing Facility. Primers 341F (5′ CCTACGGGNGGCWGCAG) and 805R (5′ GACTACHVGGGTATCTAATCC) were used to amplify the V3–V4 region of the 16S rRNA gene with a total read length of 2 × 301 bp. A bacterial mock community (Microbial Community DNA Standard, ZymoBiomics) provided by the sequencing facility was included.

### Sequence Analysis

After sequencing, the sequence reads were pre-processed, quality filtered and analysed using Quantitative Insight into Microbial Ecology (QIIME 2, v. 2019.7). Raw demultiplexed Illumina fastq sequence (Phred33 applied for quality control) and mapping files were imported into QIIME 2 for downstream processes. The manifest file was created by concatenating the forward and the reverse sequences. The DADA2 pipeline was used for denoising, quality filtering, dereplication and chimera removal [58]. Quality analysis was performed by trimming the primers and truncating the reads using the commands –p-trim-left-f 17, –p-trim-left-r 21, –p-trunc-len-f 290 and –p-trunc-len-r 210. A naive Bayes classifier was trained using the Greengenes 99% sequence similarity threshold for calling operational taxonomic units (OTUs) at the V3–V4 region of the 16S rRNA gene. Amplicon sequence variants (ASVs) from DADA2 were used for taxonomic classification at a 99% similarity threshold using QIIME 2 q2-feature-classifier plugin [59] and sample taxonomic composition, and structure was visualised using QIIME 2 bar plot and plotted in R version 3.6.3 (R core Team, 2020, https://www.R-project.org/). The core-metrics-phylogenetic pipeline was used to construct the phylogenetic tree. A rarefaction curve was used to assess adequate sampling of the microbial communities. Based on the rarefaction curve, the overall alpha and beta diversity analyses were performed at a sampling depth of 6120, and at 1000 upon filtering out the *Wolbachia* reads, to avoid biases using the q2-diversity plugin. We estimated the alpha diversity among the four groups of samples using Shannon diversity index and Pielou’s evenness. Beta diversity was assessed using weighted UniFrac distance (phylogenetic relationships and relative abundance) and Bray–Curtis distance (relative abundance) to determine the microbial community variation in the four sample groups (Dd, FliesDdW, FliesDd and Flies) with pairwise comparisons (PERMANOVA) using qiime diversity beta-group-significance in QIIME 2 (v. 2019.7). Beta diversity results were also visualised using principal coordinates analysis (PCoA) plots in R. To confirm that the *Wolbachia* ASV of our study corresponded to the *Wolbachia* previously characterised from *D. daci*, we compared it in a multiple sequence alignment using CLUSTALW together with 16S rRNA gene sequences of *w*Ddac1 and *w*Ddac2 extracted from genome reads obtained from the *Wolbachia-*positive sample *B. frauenfeldi* 485 as part of a whole genome sequencing project [28, 43] and with a cloned *Wolbachia* 16S rRNA gene (GenBank accession KC775794) sequence obtained from *B. neohumeralis* [51].

### Differential Relative Abundance Analysis

To determine whether early stages of *D. daci* parasitisation had an impact on the microbiome of the host fruit fly, we compared the relative abundance of bacterial taxa in the fruit flies parasitised by early stages of *D. daci* without detectable *Wolbachia* (FliesDd) to the unparasitised fruit flies (Flies) (Table S1). Similarly, we assessed whether parasitisation by early stages of *Wolbachia-*positive *D. daci* had an impact on the host fruit fly microbiome by comparing the FliesDd samples to the FliesDdW samples (Table S1). For these comparisons, we used the original taxonomic assignments of ASVs (at 99% identity) with the *Wolbachia* reads excluded. OTU datasets generated in QIIME and summarised at genus level were imported into Phyloseq for downstream analysis. The differential relative abundance was then performed in edgeR [60].

## Results

### Sequence Read Analysis

The 101 sequenced 16S rRNA gene amplicon libraries (Table S1) included 17 *D. daci* male pupae (Dd), 30 fruit flies parasitised by *Wolbachia*-positive *D. daci* (FliesDdW), 19 fruit flies parasitised by *D. daci* without detectable *Wolbachia* (FliesDd) and 35 unparasitised fruit flies (Flies) across seven tephritid species (*B. bryoniae*, *B. decurtans*, *B. frauenfeldi*, *B. neohumeralis*, *B. tryoni*, *D. axanus* and *Z. strigifinis*). After quality control and filtering, we obtained a total of 2,274,402 sequence reads, with a mean sequence read number of 22,519 per sample (between 42 and 120,845 sequence reads per sample). After normalising the sequence read number at a sampling depth of 6120 to minimise biases, we excluded one fruit fly specimen that contained fewer than 6120 sequences (one *D. axanus* Flies sample with 42 sequence reads) from the subsequent analysis (Table S1). A total of 1808 ASVs were identified in this study (Table S2).

### Bacterial Community of *D. daci*

**Fig. 2:**
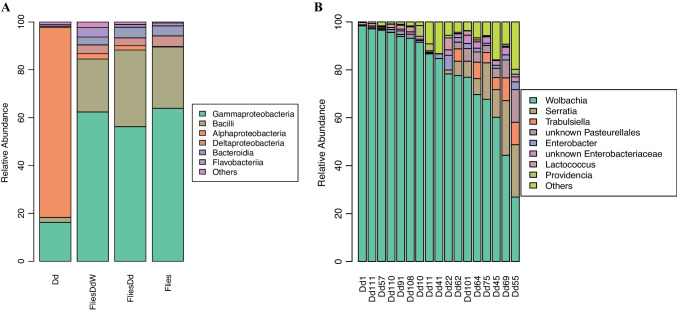
Relative abundance of bacterial taxa in *Dipterophagus daci.* **A** Barplot of the relatively abundant bacterial classes in the four categories of samples: *D. daci* male pupae (Dd), fruit flies parasitised by early stages of *Wolbachia*-positive *D. daci* (FliesDdW), fruit flies parasitised by early stages of *D. daci* without detectable *Wolbachia* (FliesDd) and unparasitised fruit flies (Flies); **B** barplot of the relatively abundant bacterial genera in the 17 *D. daci* pupae samples. The highest available classification was used for taxa with no assigned genus

The bacterial community of *D. daci* pupae was dominated by the class Alphaproteobacteria, accounting for 79.2% of the total sequence reads (Fig. 2a, Table S3)*.* Other classes include Gammaproteobacteria (16.2%), Bacilli (2.1%), Deltaproteobacteria (0.6%), Flavobacteria (0.66%), Bacteroidia and various other classes with a combined relative abundance of < 1% (Fig. 2a, Table S3)*.* The ASV with the highest relative abundance was one *Wolbachia* 16S rRNA gene sequence accounting for 78.7% of all sequence reads and was present in all 17 *D. daci* pupae (Fig. 2b, Table S4). The 16S rRNA gene sequences from *w*Ddac1 and *w*Ddac2 obtained from a previous whole genome sequencing project did not vary in the V3–V4 region (402 bp) and were identical to the dominant *Wolbachia* ASV obtained in this study (Fig. S1). An additional 15 *Wolbachia* ASVs, all singletons, consisted of sequences with up to 2 mismatches to the dominant ASV. The *Wolbachia* 16S rRNA gene sequence previously obtained in a molecular cloning experiment from the *Wolbachia*-positive *B. neohumeralis* was also identical to the *w*Ddac1 and *w*Ddac2 16S rRNA gene sequences albeit in another region (349 bp) (Fig. S1) further confirming that the two *Wolbachia* strains cannot be differentiated in the V3–V4 region. Other genera that were relatively abundant included *Serratia* (5.6%), *Trabulsiella* (2.4%), *Enterobacter* (1.6%), one unknown Pasteurellales ASV (2.4%), one unknown Enterobacteriaceae ASV (1.4%) and *Lactococcus* (1.09%) (Fig. 2b, Table S4).

**Fig. 3:**
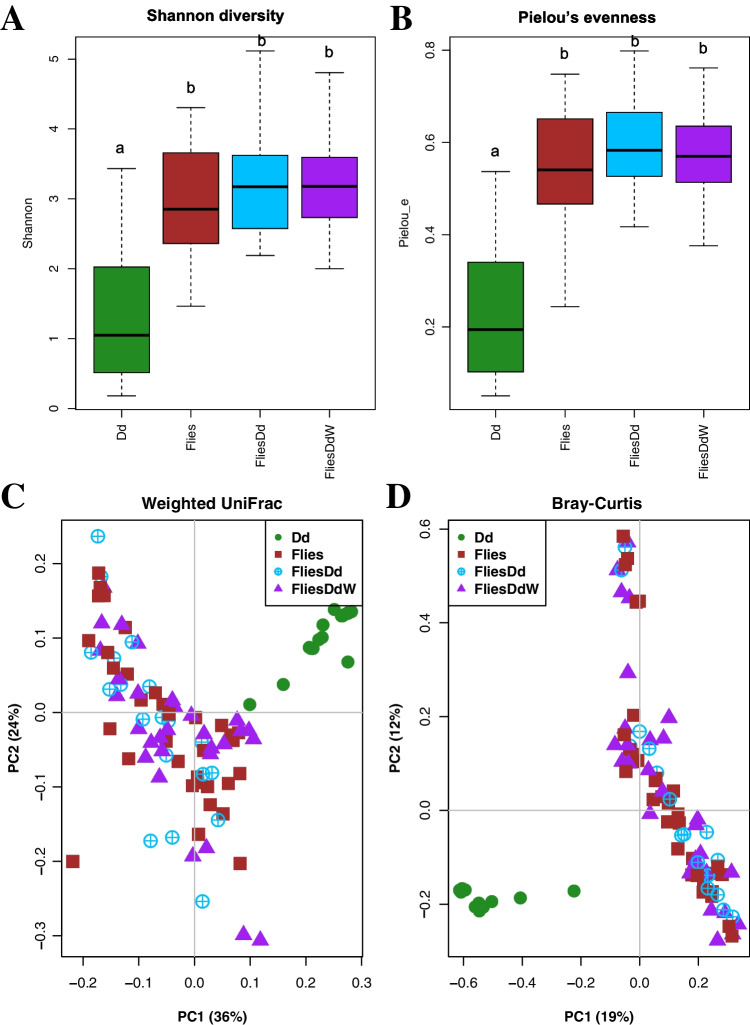
Alpha and beta diversity analysis of *Dipterophagus daci* male pupae (Dd), unparasitised fruit flies (Flies), fruit flies parasitised by early stages of *D. daci* without detectable *Wolbachia* (FliesDd) and fruit flies parasitised by early stages of *Wolbachia*-positive *D. daci* (FliesDdW). **A** Shannon diversity, **B** Pielou’s evenness, **C** weighted UniFrac and **D** Bray–Curtis principle coordinate analysis (PCoA) plots. Different letters indicate significant differences in Kruskal–Wallis comparisons (*p* < 0.05)

Alpha diversity analysis revealed low Shannon diversity and Pielou’s evenness indices in *D. daci*, whereas both indices were higher for flies (Kruskal–Wallis, *p* < 0.05, Fig. 3a and b, Table S5). Beta diversity analysis of bacterial communities using weighted UniFrac and Bray–Curtis PCoAs showed that *D. daci* bacterial communities clustered separately from those of the host fruit flies (Fig. 3c and d, Table S6 and S7). PERMANOVA analyses based on Bray–Curtis results also revealed the distinct clustering of *D. daci* bacterial communities from those of host fruit flies (Table 1).

**Table 1:** Summary of PERMANOVA results assessing pairwise beta diversity metrics differences between groups of samples: *Dipterophagus daci* male pupae (Dd), fruit flies parasitised by early stages of *Wolbachia*-positive *D. daci* (FliesDdW), fruit flies parasitised by early stages of *D. daci* without detectable *Wolbachia* (FliesDd) and unparasitised fruit flies (Flies). Comparisons that are significantly different are shown in bold

PERMANOVA			Weighted UniFrac		Bray–Curtis	
	Sample size	Permutations	pseudo-F	*p* Value	pseudo-F	*p* Value
Flies-Dd	51	999	41.014	**0.001**	18.898	**0.001**
FliesDd-Dd	36	999	47.850	**0.001**	22.044	**0.001**
FliesDdW-Dd	47	999	39.167	**0.001**	19.437	**0.001**
Flies- FliesDd	53	999	0.688	0.598	0.904	0.577
Flies-FliesDdW	64	999	0.708	0.615	1.058	0.341
FliesDd-FliesDdW	49	999	1.329	0.236	1.103	0.307

### Comparison of Bacterial Communities Among Fruit Fly Species

**Fig. 4:**
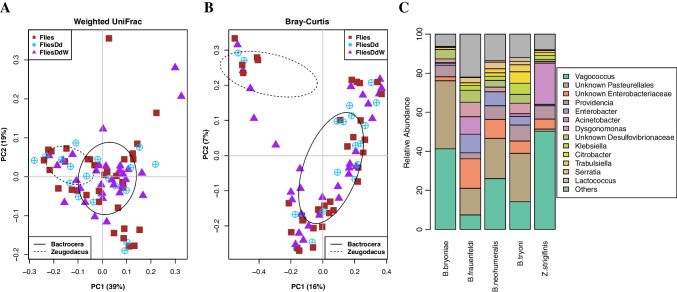
Analyses of fruit fly samples groups. **A** Weighted UniFrac and **B** Bray–Curtis beta diversity principle coordinate analysis (PCoA) plots to visualise the clustering and similarity of the fruit fly sample groups. The ellipses drawn based on the standard deviation show the clustering of the *Bactrocera* and *Zeugodacus* samples. **C** Bar plot of the most common bacterial genera in the host fruit flies *Bactrocera bryoniae*, *Bactrocera frauenfeldi*, *Bactrocera neohumeralis*, *Bactrocera tryoni* and *Zeugodacus strigifinis*. Analysis was performed on fruit fly specimens from all sample groups including unparasitised fruit flies (Flies), fruit flies parasitised by early stages of *Dipterophagus daci* without detectable *Wolbachia* (FliesDd) and fruit flies parasitised by early stages of *Wolbachia*-positive *D. daci* (FliesDdW)

The weighted UniFrac analysis showed no distinct clustering pattern in the fruit fly species (Fig. 4a, Table S8). However, Bray–Curtis PCoA revealed a distinct separation in the bacterial communities of *Z. strigifinis* and the remaining six fruit fly species (Fig. 4b, Table S9). Therefore, the fruit fly bacterial communities of *Z. strigifinis, B. bryoniae*, *B. frauenfeldi*, *B. neohumeralis* and *B. tryoni* were investigated to determine the differences in relative abundances (*B. decurtans* and *D. axanus* were not included due to low sample numbers). Prior to this, the alpha diversity analysis of the five fruit fly species was performed. Both Shannon diversity and Pielou’s evenness indices revealed a significant difference in bacterial communities of *B. frauenfeldi* and *Z. strigifinis* compared to other fruit fly species (Kruskal–Wallis, *p* < 0.05, Fig. S2, Table S10).

Bacterial genera with the highest abundance in all fruit flies were *Vagoccoccus* (27.8%), one unknown Pasteurellales ASV (19.0%), one unknown Enterobacteriacea ASV (7.7%), *Acinetobacter* (6.4%), *Providencia* (6.2%), *Enterobacter* (4.6%), one unknown Desulfovibrionaceae ASV (3.9%), *Dysgonomonas* (3.6%), *Klebsiella* (2.5%), *Citrobacter* (2.1%), *Serratia* (1.7%), *Trabulsiella* (1.6%), *Lactococcus* (0.63%) and others (all remaining bacteria combined, 12.4%) (Fig. 4c, Table S11). Bar plots representing the diversity of the fruit fly bacterial communities revealed variability in the relative abundance of bacteria (Fig. 4c). The most striking difference was the low relative abundance of the one unknown Pasturellales ASV in *Z. strigifinis* (1%) compared to the other fruit fly species which contained this bacterium at relative abundances ranging from 13 to 35% (Fig. 4c, Table S11). Interestingly, *Acinetobacter* bacteria were relatively more abundant in *Z. strigifinis* (21%) compared to *B. bryoniae* (0.2%), *B. frauenfeldi* (8.9%), *B. neohumeralis* (0.01%) and *B. tryoni* (1.4%) (Fig. 4c, Table S11). PERMANOVA pairwise analyses based on both weighted UniFrac and Bray–Curtis results showed significant differences (*p* < 0.05, PERMANOVA) in bacterial communities of *B. frauenfeldi* compared to the other fruit fly species (Table 2). This could be attributed to the one unknown Enterobacteriaceae ASV that had a relative abundance of 15.1% in *B. frauenfeldi*, while the other fruit fly species had relative abundances of this unknown Enterobacteriaceae ASV ranging from 2.05 to 9.7% (Fig. 4c, Table S11). No significant differences were observed in comparisons among the bacterial communities of *B. bryoniae, B. neohumeralis* and *B. tryoni* (Table 2)*.*

**Table 2:** Summary of PERMANOVA results assessing beta diversity metrics pairwise differences between host fruit fly species: *Bactrocera bryoniae*, *Bactrocera frauenfeldi*, *Bactrocera neohumeralis*, *Bactrocera tryoni* and *Zeugodacus strigifinis* (*Bactrocera decurtans* and *Dacus axanus* were not included due to low sample numbers). Comparisons that are significantly different are shown in bold

PERMANOVA			Weighted UniFrac		Bray–Curtis	
	Sample size	Permutations	pseudo-F	*p* Value	pseudo-F	*p* Value
*B. bryoniae*-*B. frauenfeldi*	15	999	4.967	**0.011**	3.026	**0.002**
*B. bryoniae-B. neohumeralis*	26	999	1.348	0.246	1.548	0.068
*B. bryoniae-Z. strigifinis*	15	999	5.532	**0.005**	15.058	**0.001**
*B. bryoniae-B. tryoni*	36	999	2.398	0.065	1.778	**0.017**
*B. frauenfeldi-B. neohumeralis*	33	999	7.010	**0.001**	2.431	**0.002**
*B. frauenfeldi-Z. strigifinis*	22	999	13.883	**0.001**	7.418	**0.001**
*B. frauenfeldi-B. tryoni*	43	999	3.863	**0.004**	1.851	**0.004**
*B. neohumeralis-Z. strigifinis*	33	999	9.187	**0.001**	11.844	**0.001**
*B. neohumeralis-B. tryoni*	54	999	1.790	0.134	1.324	0.113
*Z. strigifinis-B. tryoni*	43	999	14.541	**0.001**	10.172	**0.001**

### Influence of Early Stages of *D. daci* Parasitisation on Bacterial Communities in Fruit Fly Hosts

**Fig. 5:**
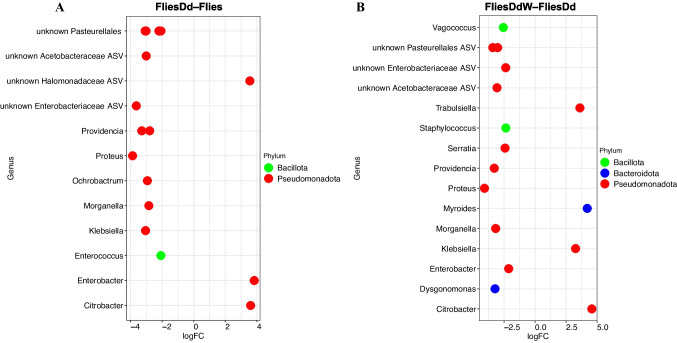
Scatter plot of the bacterial taxa with differential relative abundance in **A** fruit flies parasitised by early stages of *Dipterophagus daci* without detectable *Wolbachia* (FliesDd) compared to unparasitised fruit (Flies) and in **B** fruit flies parasitised by early stages of *Wolbachia-*positive *Dipterophagus daci* (FliesDdW) compared to fruit flies parasitised by early stages of *D. daci* without detectable *Wolbachia* (FliesDd). A log fold change of logFC > 0 indicates that the abundance of the genera increased, whereas logFC < 0 indicates that the abundance of the genera decreased. The taxa with significantly different relative abundances are coloured by phylum

A comparison of the fruit flies parasitised by early stages of *D. daci* without detectable *Wolbachia* (FliesDd) and unparasitised flies (Flies) was performed to determine the impact of early *D. daci* parasitisation on the fruit fly bacterial diversity. The OTU datasets used were retrieved from QIIME and summarised to genus level. This comparison revealed an impact of early parasitisation by *D. daci* on the relative abundance of Pseudomonadota (formerly Proteobacteria) and Bacillota (formerly Firmicutes) in fruit flies (Fig. 5a, Table S12). The relative abundance of nine bacterial genera including *Proteus*, one unknown Enterobacteriaceae ASV, *Klebsiella*, one unknown Acetobacteriacea ASV, *Ochrobactrum*, *Morganella*, *Providencia*, three unknown Pasteurellales ASVs and *Enterococcus* were increased in FliesDd, while three bacterial genera (*Enterobacter, Citrobacter* and one unknown Halomonadacea ASV) decreased in FliesDd (Fig. 5a, Table S12).

Similarly, we compared the relative abundance of bacterial taxa between flies parasitised by *D. daci* with and without detectable *Wolbachia*. Sequences used in this analysis were corrected for *Wolbachia* and normalised to a sequencing depth of 1000, based on the minimum number of reads after excluding *Wolbachia*. We found that the relative abundance of 11 genera comprising *Proteus*, *Providencia*, *Dysgonomonas*, *Morganella*, one unknown Acetobacteriacea ASV, two unknown Pasteurellales ASVs, *Vagococcus*, *Serratia*, one unknown Enterobacteriaceae ASV,* Staphylococcus* and *Enterobacter* were decreased in FliesDd*,* while the relative abundances of *Klebsiella*, *Trabulsiella*, *Myroides* and *Citrobacter* were increased (Fig. 5b, Table S13).

## Discussion

We used 16S rRNA gene amplicon sequencing to characterise the bacterial communities involved in the interactions between tephritid fruit flies and the strepsipteran endoparasitoid *D. daci*. With this we have performed, according to our knowledge, the first comprehensive characterisation of bacterial communities in a species of the endoparasitic insect order Strepsiptera [23]. The bacterial communities of *D. daci* were dominated by *Wolbachia*; however, this dominance was not observed in fruit flies parasitised by early stages of *Wolbachia*-positive *D. daci* (and *Wolbachia* was completely absent in flies not parasitised by *D. daci*), supporting previous findings that *D. daci* is the host of *Wolbachia* in this host-parasitoid interaction [28]. We found that the bacterial communities of *D. daci* are not as diverse but distinct when compared to the more diverse bacterial communities of the fruit fly hosts. Furthermore, early stages of *D. daci* parasitisation and presence of *Wolbachia* in *D. daci* altered the microbiome of parasitised fruit flies. We also found that the bacterial communities of *Z. strigifinis* were distinct from the bacterial communities of the *Bactrocera* species and this may be linked to their different ecologies, with *Z. strigifinis* developing in cucurbit flowers, whereas the analysed *Bactrocera* species develop in fruit [53, 61].

### *Dipterophagus daci* Has a Less Diverse Microbiome

The most abundant bacterial phylum in *D. daci* was Pseudomonadota comprising 96.2% of the total bacterial sequence reads, followed by Bacillota and Bacteroidota at a substantially lower relative abundance. A high relative abundance of Pseudomonadota and Bacillota has previously been detected in fruit flies [54, 62] and other insect species [55, 63]; however, the relative abundance of Pseudomonadota in *D. daci* pupae found in our study was generally higher and mostly just consisted of *Wolbachia*. This indicates that bacterial communities in *D. daci* are not very diverse, which is perhaps due to its parasitic life cycle. The strepsipteran *D. daci* and all other Strepsiptera are almost fully endoparasitic in their host and depend exclusively on the host for nourishment [24, 26, 33]. The high presentation of Pseudomonadota in *D. daci* was due to *Wolbachia*, a member of the Alphaproteobacteria, in combination with Gammaproteobacteria, Bacilli, Deltaproteobacteria, Bacteroidia and Flavobacteriia at substantially lower relative abundance. It needs to be noted that we were only able to characterise the bacterial communities of *D. daci* in isolation from its host by carefully dissecting pupae out of the cephalotheca extruding from the abdomen of parasitised fruit flies, followed by surface treatment to minimise contamination. We do not know how bacterial communities in *D. daci* change throughout its development. Given the endoparasitic life cycle of *D. daci*, it is likely that exposure to environmental bacteria is limited, which could impact the observed low levels of bacterial diversity in *D. daci*. Therefore, bacterial symbionts detected in *D. daci* pupae are either maternally inherited or horizontally acquired from the host fly or from the environment during the short period that planidial larvae search for new hosts. It is perhaps less likely that bacteria acquired by adult *D. daci* males are then paternally transmitted. We did not obtain free living males of *D. daci* as they would require different sampling techniques such as light trapping or collection of adult males emerging from parasitised flies and are, therefore, more difficult to collect than parasitised flies.

### The Microbiome of *D. daci* is Dominated by *Wolbachia* and Distinct from the Fruit Fly Hosts' Bacterial Communities

*Wolbachia* is a common maternally inherited endosymbiont of insects and other arthropods that can manipulate host reproduction to increase its prevalence in host populations [8, 64, 65]. In several host species, *Wolbachia* provides fitness benefits which can also maintain this endosymbiont in host populations [66]. For several insect species, it has been found that, when present, *Wolbachia* can dominate bacterial communities within hosts [67, 68]. Our findings of the dominance of *Wolbachi*a in bacterial communities within *D. daci* (but not in the bacterial communities within fruit flies) further confirms that the two *Wolbachia* strains first detected in fruit flies [50, 51] are actually associated with *D. daci* and had previously been detected in these fruit flies because of parasitisation by concealed early stages of *Wolbachia*-positive *D. daci* [28]. Additionally, alpha diversity analysis revealed low Shannon diversity and Pielou’s evenness values in *D. daci* bacterial communities (a consequence of the *Wolbachia* dominance) while this was not observed in fruit flies parasitised by early stages of *Wolbachia*-positive *D. daci*.

Previous analyses have found that *Wolbachia* occurs at high prevalence in *D. daci* [28], and *D. daci* is depauperate in mitogenome diversity across large parts of its geographic distribution [28, 43]. Because of maternal co-inheritance with mitochondria, *Wolbachia* may have caused a selective sweep of mitochondria due to either reproductive manipulation or beneficial host fitness effects. Previous analyses of whole genome sequenced specimens have not detected the presence of *Wolbachia* genes involved in reproductive manipulations, and, therefore, it is likely that *Wolbachia* confers a fitness benefit to *D. daci* [28], for example, by providing a key nutrient and/or supporting immunity; however, this will need further investigation. Most strepsipteran life stages are fully endoparasitic except for the free-living first instar larvae (planidia) and adult males and are therefore fully dependent on the host for nourishment [42]. The host may not always provide all the essential nutrition, and therefore endoparasitoids may form beneficial interactions with maternally inherited endosymbionts like *Wolbachia*. In the bedbug, *Cimex lectularius,* a *Wolbachia* supergroup F strain, provides B vitamins which are deficient in the bedbug’s diet [14]. Similarly, a *Wolbachia* supergroup A strain provides *D. melanogaster* with metabolic support in periods of nutritional stress [69], and *Wolbachia* supergroup B strains have been associated with synthesis of biotin and riboflavin to increase host fitness in the small brown planthopper *Laodelphax striatellus* and the brown planthopper *Nilaparvata lugens* [15]. Furthermore, *Wolbachia* supergroup A strains provide protection against pathogens such as RNA viruses in several insect species such as *Drosophila* [11, 13, 70] and mosquitoes [71, 72]. Throughout its entire development, *D. daci* is exposed to the fruit flies’ immune system and the host’s viruses. It has recently been found that the tephritid host species of *D. daci* have a very high incidence and prevalence of insect-specific RNA viruses [73] with vertical and horizontal transmission modes [74]. Future research should investigate how these viruses interact with fruit fly hosts and *D. daci*.

Furthermore, the weighted UniFrac and Bray–Curtis beta diversity analyses revealed that the bacterial community of *D. daci* was distinct from the bacterial communities of its fruit fly host species. This may be due to the phylogenetic distance between the strepsipteran and its fruit fly hosts, or the differences in host life cycle and diet. It also indicates that *D. daci* and the fruit fly hosts do not share microbiome components.

### Variable Bacterial Communities in Fruit Fly Host Species

Our analyses of the bacterial communities in fruit flies with the presence of several Enterobacteriaceae taxa (one unknown Enterobacteriaceae ASV, *Acinetobacter, Providencia*, *Enterobacter*, *Klebsiella*, *Citrobacter* and *Serratia*) confirmed their importance in tephritid fruit fly microbiomes as found in previous studies [54, 56, 75]*.* However, we also found an abundance of bacterial taxa such as *Vagoccoccus,* one unknown Pasteurellales ASV, *Trabulsiella*, one unknown Desulfovibrionaceae ASV and *Dysgonomonas*, which were different bacterial community members when compared to previous studies on tephritid fruit flies. This difference could be attributed to our sample collection and handling procedures (samples were collected in male lure traps with an insecticide and kept dry and at room temperature until identification). Additionally, for our study, we specifically selected individuals that were parasitised by early stages of *D. daci* and this could also have resulted in a sampling bias.

Tephritid fruit fly species exhibit diverse life histories and host plant preferences [52, 53, 61], and these can affect their microbiomes [53, 54, 76]. The Shannon diversity and Pielou’s evenness showed significant difference in *B. frauenfeldi* and *Z. strigifinis* bacterial communities compared to the other fruit flies. Additionally, the Bray–Curtis PCoA revealed that bacterial communities associated with *Z. strigifinis* were distinct from those of *Bactrocera* species, possibly suggesting a fly genus effect, albeit we only included one *Zeugodacus* species in our study. Furthermore, there could be a host plant effect as *Z. strigifinis* is a pest of *Cucurbitaceae* flowers while *B. tryoni*, *B. neohumeralis*, *B. frauenfeldi* and *B. bryoniae* infest fruits [53, 61]. The weighted UniFrac analysis, however, did not show any distinct clustering, suggesting that the variation between the bacterial communities of *Z. strigifinis* and the *Bactrocera* species may only be in the relative abundance of the bacterial taxa that may have similar function. This difference may be due to the unknown ASVs of Pasteurellales and *Acinetobacter*. In addition to the different bacterial communities observed in *Z. strigifinis*, PERMANOVA revealed that *B. frauenfeldi* were also different in bacterial community structure when compared to *B. bryoniae*, *B. tryoni* and *B. neohumeralis*. *Bactrocera tryoni* and *B. neohumeralis* are closely related sibling species [77]; hence, this may explain the similarity of their bacterial communities [54], while it is unclear why *B. bryoniae* grouped with these two species.

### *Dipterophagus daci* Parasitisation Alters Structure of Bacterial Communities

We observed a significant decrease in the relative abundance of nine bacterial genera in fruit flies parasitised by *D. daci* without *Wolbachia*, while three bacterial genera increased in their relative abundance, suggesting that *D. daci* parasitisation affects the relative abundance of bacterial taxa in host bacterial communities. Furthermore, despite the low relative abundance of *Wolbachia* in fruit flies parasitised by *Wolbachia*-positive *D. daci*, we found a decrease in the relative abundance of 11 bacterial genera in flies parasitised by *Wolbachia*-positive *D. daci*, while the relative abundance of four bacterial genera increased. This suggests that parasitisation by *D. daci* and presence of *Wolbachia* in *D. daci* affect bacterial communities in flies. This is in line with other research that has shown that microbes can influence host-parasite interactions [19, 29, 32, 35, 78]

## Conclusions

According to our knowledge, our study is the first comprehensive characterisation of the bacterial communities of a strepsipteran using a next generation amplicon sequencing approach. We demonstrated that bacterial communities of *D. daci* are not very diverse, dominated by *Wolbachia* and distinct from those of its host fruit fly species, and this could be attributed to the differences in host life cycles, life histories and phylogeny. Further studies should investigate the role of the two *Wolbachia* strains in *D. daci*, in particular as it is clear from previous genome analyses that they lack the capacity to manipulate host reproduction yet have an overall high prevalence in *D. daci* [28]. Furthermore, we observed variability in the relative abundance of bacterial taxa across fruit fly species, irrespective of parasitisation by *D. daci*, suggesting that phylogeny, host plant preference and host plant use play a role in shaping bacterial communities in fruit flies [54]. In addition, early stages of *D. daci* parasitisation affected the relative abundance of bacteria in microbial communities of host fruit flies. Hence, parasitisation can also shape the microbiome of insects and should therefore be considered in host-microbiome studies.

## Supplementary Information

Below is the link to the electronic supplementary material.

Supplementary file1 (DOCX 176 KB)

Supplementary file2 (XLSX 979 KB)

## Data Availability

The raw bacterial 16S rRNA gene sequence reads have been deposited in GenBank Sequence Read Archive ﻿(GenBank accession SAMN26586984- SAMN26587084).
