# Supplementary material for: Bacterial Communities Are Less Diverse in a Strepsipteran Endoparasitoid than in Its Fruit Fly Hosts and Dominated by Wolbachia
Source: Microb Ecol. 2023 Apr 27;86(3):2120–32. doi: 10.1007/s00248-023-02218-6 (PMC10497669; doi:10.1007/s00248-023-02218-6)
Supplement: Supplementary file 1 — Supplementary file1 (DOCX 176 KB) [file 248_2023_2218_MOESM1_ESM.docx]

Electronic Supplementary Material Figures S1 and S2 (Towett-Kirui et al., Microbial Ecology)

KC775794 ------------------------------------------------------------ 0

BfraWolb16S_a AAATTTGAGAGTTTGATCCTGGCTCAGAATGAACGCTGGCGGCAGGCCTAACACATGCAA 60

BfraWolb16S_b AAATTTGAGAGTTTGATCCTGGCTCAGAATGAACGCTGGCGGCAGGCCTAACACATGCAA 60

a41cfc760144d8cdf9909c2681abe105 ------------------------------------------------------------ 0

KC775794 ------------------------------------------------------------ 0

BfraWolb16S_a GTCGAACGGAGTTATATTGTAGCTTGCTATGGTGTAACTTAGTGGCAGACGGGTGAGTAA 120

BfraWolb16S_b GTCGAACGGAGTTATATTGTAGCTTGCTACGATATAACTTAGTGGCAGACGGGTGAGTAA 120

a41cfc760144d8cdf9909c2681abe105 ------------------------------------------------------------ 0

KC775794 ------------------------------------------------------------ 0

BfraWolb16S_a TGTATAGGAATCTACCTAGTAGTACGGAATAATTGTTGGAAACGGCAACTAATACCGTAT 180

BfraWolb16S_b TGTATAGGAATCTACCTAGTAGTACGGAATAATTGTTGGAAACGGCAACTAATACCGTAT 180

a41cfc760144d8cdf9909c2681abe105 ------------------------------------------------------------ 0

KC775794 ------------------------------------------------------------ 0

BfraWolb16S_a ACGCCCTACGGGGGAAAAATTTATTGCTATTAGATGAGCCTATATTAGATTAGCTAGTTG 240

BfraWolb16S_b ACGCCCTACGGGGGAAAAATTTATTGCTATTAGATGAGCCTATATTAGATTAGCTAGTTG 240

a41cfc760144d8cdf9909c2681abe105 ------------------------------------------------------------ 0

KC775794 ------------------------------------------------------------ 0

BfraWolb16S_a GTGGAGTAATAGCCTACCAAGGCAATGATCTATAGCTGATCTGAGAGGATGATCAGCCAC 300

BfraWolb16S_b GTGGAGTAATAGCCTACCAAGGCAATGATCTATAGCTGATCTGAGAGGATGATCAGCCAC 300

a41cfc760144d8cdf9909c2681abe105 ------------------------------------------------------------ 0

KC775794 ------------------------------------------------------------ 0

BfraWolb16S_a ACTGGAACTGAGATACGGTCCAGACTCCTACGGGAGGCAGCAGTGGGGAATATTGGACAA 360

BfraWolb16S_b ACTGGAACTGAGATACGGTCCAGACTCCTACGGGAGGCAGCAGTGGGGAATATTGGACAA 360

a41cfc760144d8cdf9909c2681abe105 -------------------------------------------TGGGGAATATTGGACAA 17

KC775794 ------------------------------------------------------------ 0

BfraWolb16S_a TGGGCGAAAGCCTGATCCAGCCATGCCGCATGAGTGAAGAAGGCCTTTGGGTTGTAAAGC 420

BfraWolb16S_b TGGGCGAAAGCCTGATCCAGCCATGCCGCATGAGTGAAGAAGGCCTTTGGGTTGTAAAGC 420

a41cfc760144d8cdf9909c2681abe105 TGGGCGAAAGCCTGATCCAGCCATGCCGCATGAGTGAAGAAGGCCTTTGGGTTGTAAAGC 77

KC775794 ------------------------------------------------------------ 0

BfraWolb16S_a TCTTTTAGTGAGGAAGATAATGACGGTACTCACAGAAGAAGTCCTGGCTAACTCCGTGCC 480

BfraWolb16S_b TCTTTTAGTGAGGAAGATAATGACGGTACTCACAGAAGAAGTCCTGGCTAACTCCGTGCC 480

a41cfc760144d8cdf9909c2681abe105 TCTTTTAGTGAGGAAGATAATGACGGTACTCACAGAAGAAGTCCTGGCTAACTCCGTGCC 137

KC775794 ------------------------------------------------------------ 0

BfraWolb16S_a AGCAGCCGCGGTAATACGGAGAGGGCTAGCGTTATTCGGAATTATTGGGCGTAAAGGGCG 540

BfraWolb16S_b AGCAGCCGCGGTAATACGGAGAGGGCTAGCGTTATTCGGAATTATTGGGCGTAAAGGGCG 540

a41cfc760144d8cdf9909c2681abe105 AGCAGCCGCGGTAATACGGAGAGGGCTAGCGTTATTCGGAATTATTGGGCGTAAAGGGCG 197

KC775794 ------------------------------------------------------------ 0

BfraWolb16S_a CGTAGGCGGATTAGTAAGTTAAAAGTGAAATCCCAAGGCTCAACCTTGGAATTGCTTTTA 600

BfraWolb16S_b CGTAGGCGGATTAGTAAGTTAAAAGTGAAATCCCAAGGCTCAACCTTGGAATTGCTTTTA 600

a41cfc760144d8cdf9909c2681abe105 CGTAGGCGGATTAGTAAGTTAAAAGTGAAATCCCAAGGCTCAACCTTGGAATTGCTTTTA 257

KC775794 ------------------------------------------------------------ 0

BfraWolb16S_a AAACTGCTAATCTAGAGATTGAAAGAGGATAGAGGAATTCCTAGTGTAGAGGTGAAATTC 660

BfraWolb16S_b AAACTGCTAATCTAGAGATTGAAAGAGGATAGAGGAATTCCTAGTGTAGAGGTGAAATTC 660

a41cfc760144d8cdf9909c2681abe105 AAACTGCTAATCTAGAGATTGAAAGAGGATAGAGGAATTCCTAGTGTAGAGGTGAAATTC 317

KC775794 ------------------------------------------------------------ 0

BfraWolb16S_a GTAAATATTAGGAGGAACACCAGTGGCGAAGGCGTCTATCTGGTTCAAATCTGACGCTGA 720

BfraWolb16S_b GTAAATATTAGGAGGAACACCAGTGGCGAAGGCGTCTATCTGGTTCAAATCTGACGCTGA 720

a41cfc760144d8cdf9909c2681abe105 GTAAATATTAGGAGGAACACCAGTGGCGAAGGCGTCTATCTGGTTCAAATCTGACGCTGA 377

KC775794 ------------------------------------------------------------ 0

BfraWolb16S_a GGCGCGAAGGCGTGGGGAGCAAACAGGATTAGATACCCTGGTAGTCCACGCTGTAAACGA 780

BfraWolb16S_b GGCGCGAAGGCGTGGGGAGCAAACAGGATTAGATACCCTGGTAGTCCACGCTGTAAACGA 780

a41cfc760144d8cdf9909c2681abe105 GGCGCGAAGGCGTGGGGAGCAAACA----------------------------------- 402

KC775794 ------------------------------------------------------------ 0

BfraWolb16S_a TGAATGTTAAATATGGGAAGTTTTACTTTCTGTATTACAGCTAACGCGTTAAACATTCCG 840

BfraWolb16S_b TGAATGTTAAATATGGGAAGTTTTACTTTCTGTATTACAGCTAACGCGTTAAACATTCCG 840

a41cfc760144d8cdf9909c2681abe105 ------------------------------------------------------------ 402

KC775794 ------------------------------------------------------------ 0

BfraWolb16S_a CCTGGGGACTACGGTCGCAAGATTAAAACTCAAAGGAATTGACGGGGACCCGCACAAGCG 900

BfraWolb16S_b CCTGGGGACTACGGTCGCAAGATTAAAACTCAAAGGAATTGACGGGGACCCGCACAAGCG 900

a41cfc760144d8cdf9909c2681abe105 ------------------------------------------------------------ 402

KC775794 ------------------------------------------------------------ 0

BfraWolb16S_a GTGGAGCATGTGGTTTAATTCGATGCAACGCGAAAAACCTTACCACTCCTTGACATGGAA 960

BfraWolb16S_b GTGGAGCATGTGGTTTAATTCGATGCAACGCGAAAAACCTTACCACTCCTTGACATGGAA 960

a41cfc760144d8cdf9909c2681abe105 ------------------------------------------------------------ 402

KC775794 -------------------------------------------------GTGTTGCATGG 11

BfraWolb16S_a ATTATACCTATTCGAAGGGATAGGGTCGGTTCGGCCGGGTTTCACACAGGTGTTGCATGG 1020

BfraWolb16S_b ATTATACCTATTCGAAGGGATAGGGTCGGTTCGGCCGGGTTTCACACAGGTGTTGCATGG 1020

a41cfc760144d8cdf9909c2681abe105 ------------------------------------------------------------ 402

KC775794 CTGTCGTCAGCTCGTGTCGTGAGATGTTGGGTTAAGTCCCGCAACGAGCGCAACCCTCAT 71

BfraWolb16S_a CTGTCGTCAGCTCGTGTCGTGAGATGTTGGGTTAAGTCCCGCAACGAGCGCAACCCTCAT 1080

BfraWolb16S_b CTGTCGTCAGCTCGTGTCGTGAGATGTTGGGTTAAGTCCCGCAACGAGCGCAACCCTCAT 1080

a41cfc760144d8cdf9909c2681abe105 ------------------------------------------------------------ 402

KC775794 CCTTAGTTACCATCAGGTAATGCTGGGGACTTTAAGGAAACTGCCAGTGATAAACTGGAG 131

BfraWolb16S_a CCTTAGTTACCATCAGGTAATGCTGGGGACTTTAAGGAAACTGCCAGTGATAAACTGGAG 1140

BfraWolb16S_b CCTTAGTTACCATCAGGTAATGCTGGGGACTTTAAGGAAACTGCCAGTGATAAACTGGAG 1140

a41cfc760144d8cdf9909c2681abe105 ------------------------------------------------------------ 402

KC775794 GAAGGTGGGGATGATGTCAAGTCATCATGGCCCTTATGGAGTGGGCTACACACGTGCTAC 191

BfraWolb16S_a GAAGGTGGGGATGATGTCAAGTCATCATGGCCCTTATGGAGTGGGCTACACACGTGCTAC 1200

BfraWolb16S_b GAAGGTGGGGATGATGTCAAGTCATCATGGCCCTTATGGAGTGGGCTACACACGTGCTAC 1200

a41cfc760144d8cdf9909c2681abe105 ------------------------------------------------------------ 402

KC775794 AATGGTGGCTACAATGGGCTGCAAAGTCGCGAGGCTAAGCCAATCCCTTAAAAGCCATCT 251

BfraWolb16S_a AATGGTGGCTACAATGGGCTGCAAAGTCGCGAGGCTAAGCCAATCCCTTAAAAGCCATCT 1260

BfraWolb16S_b AATGGTGGCTACAATGGGCTGCAAAGTCGCGAGGCTAAGCTAATCCCTTAAAAGCCATCT 1260

a41cfc760144d8cdf9909c2681abe105 ------------------------------------------------------------ 402

KC775794 CAGTTCGGATTGTACTCTGCAACTCGAGTGCATGAAGTTGGAATCGCTAGTAATCGTGGA 311

BfraWolb16S_a CAGTTCGGATTGTACTCTGCAACTCGAGTGCATGAAGTTGGAATCGCTAGTAATCGTGGA 1320

BfraWolb16S_b CAGTTCGGATTGTACTCTGCAACTCGAGTGCATGAAGTTGGAATCGCTAGTAATCGTGGA 1320

a41cfc760144d8cdf9909c2681abe105 ------------------------------------------------------------ 402

KC775794 TCAGCACGCCACGGTGAATACGTTCTCGGGTCTTGTACACACTGCCCGTCACGCCATGGG 371

BfraWolb16S_a TCAGCACGCCACGGTGAATACGTTCTCGGGTCTTGTACACACTGCCCGTCACGCCATGGG 1380

BfraWolb16S_b TCAGCACGCCACGGTGAATACGTTCTCGGGTCTTGTACACACTGCCCGTCACGCCATGGG 1380

a41cfc760144d8cdf9909c2681abe105 ------------------------------------------------------------ 402

KC775794 AATTGGTT---------------------------------------------------- 379

BfraWolb16S_a AATTGGTTTCACTCGAAGCTAACGACCTAACCGCAAGGAGGGAGTTATTTAAAGTGGGAT 1440

BfraWolb16S_b AATTGGTTTCACTCGAAGCTAACGACCTAACCGCAAGGAGGGAGTTATTTAAAGTGGGAT 1440

a41cfc760144d8cdf9909c2681abe105 ------------------------------------------------------------ 402

KC775794 ------------------------------------------------------------ 379

BfraWolb16S_a CGGTGACTGGGGTGAAGTCGTAACAAGGTAGCAGTAGGGGAATCTGCAGCTGGATTACCT 1500

BfraWolb16S_b CGGTGACTGGGGTGAAGTCGTAACAAGGTAGCAGTAGGGGAATCTGCAGCTGGATTACCT 1500

a41cfc760144d8cdf9909c2681abe105 ------------------------------------------------------------ 402

KC775794 -- 379

BfraWolb16S_a CC 1502

BfraWolb16S_b CC 1502

a41cfc760144d8cdf9909c2681abe105 -- 402

**Fig S1:** Multiple sequence alignment of 16S rRNA gene sequences of the dominant Wolbachia ASV from this study (bottom row), 16S rRNA gene sequences of wDdac1 (BfraWolb16S_a) and wDdac2 (BfraWolb16S_b) from a previous whole genome sequencing project (Towett-Kirui et al. 2021) and a cloned Wolbachia 16S rRNA gene sequence obtained from B. neohumeralis (KC775794) (Morrow et al. 2015). The three sequences are identical across the available regions.

**Fig. S2** Alpha diversity indices (Shannon diversity and Pielou’s evenness) of fruit fly species Bactrocera bryoniae, Bactrocera frauenfeldi, Bactrocera neohumeralis, Bactrocera tryoni and Zeugodacus strigifinis. Analysis performed on fruit fly specimens from all sample groups including unparasitised fruit flies (Flies), fruit flies parasitised by early stages of Dipterophagus daci without detectable Wolbachia (FliesDd) and fruit flies parasitised by early stages of Wolbachia-positive D. daci (FliesDdW). Different letters indicate significant differences in Kruskal-Wallis comparisons (p<0.05).
